# Impact of Pneumococcal Conjugate Vaccine Administration in Pediatric Older Age Groups in Low and Middle Income Countries: A Systematic Review

**DOI:** 10.1371/journal.pone.0135270

**Published:** 2015-09-02

**Authors:** Kimberly Bonner, Emily Welch, Kate Elder, Jennifer Cohn

**Affiliations:** 1 Médecins Sans Frontières, Access Campaign, Geneva, Switzerland; 2 Médecins Sans Frontières, Access Campaign, New York, New York, United States of America; 3 School of Public Health, Boston University, Boston, Massachusetts, United States of America; 4 Division of Infectious Diseases, University of Pennsylvania School of Medicine, Philadelphia, Pennsylvania, United States of America; Public Health England, UNITED KINGDOM

## Abstract

**Introduction:**

Pneumococcal conjugate vaccine (PCV) is included in the World Health Organization’s routine immunization schedule and is recommended by WHO for vaccination in high-risk children up to 60 months. However, many countries do not recommend vaccination in older age groups, nor have donors committed to supporting extended age group vaccination. To better inform decision-making, this systematic review examines the direct impact of extended age group vaccination in children over 12 months in low and middle income countries.

**Methods:**

An *a priori* protocol was used. Using pre-specified terms, a search was conducted using PubMed, LILACS, Cochrane Infectious Diseases Group Specialized Register, Cochrane Central Register of Controlled Trials, CAB Abstracts, clinicaltrials.gov and the International Symposium on Pneumococci and Pneumococcal Diseases abstracts. The primary outcome was disease incidence, with antibody titers and nasopharyngeal carriage included as secondary outcomes.

**Results:**

Eighteen studies reported on disease incidence, immune response, and nasopharyngeal carriage. PCV administered after 12 months of age led to significant declines in invasive pneumococcal disease. Immune response to vaccine type serotypes was significantly higher for those vaccinated at older ages than the unimmunized at the established 0.2ug/ml and 0.35ug/ml thresholds. Vaccination administered after one year of age significantly reduced VT carriage with odds ratios ranging from 0.213 to 0.69 over four years. A GRADE analysis indicated that the studies were of high quality.

**Discussion:**

PCV administration in children over 12 months leads to significant protection. The direct impact of PCV administration, coupled with the large cohort of children missed in first year vaccination, indicates that countries should initiate or expand PCV immunization for extended age group vaccinations. Donors should support implementation of PCV as part of delayed or interrupted immunization for older children. For countries to effectively implement extended age vaccinations, access to affordably-priced PCV is critical.

## Introduction

Responsible for 1.3 million deaths annually in children under five years, pneumonia remains a major cause of mortality, particularly in low and middle income countries[1]. Most deaths from severe pneumonia cases (33%) are caused by *S*. *pneumoniae*[1]. Over 90 serotypes have been identified, with distribution varying by geography[2].

Three vaccines protect against *S*. *pneumoniae* serotypes. Pneumococcal conjugate vaccine (PCV) 10 (GlaxoSmithKline, Belgium) contains antigens for serotypes 1, 4, 5, 6B, 7F, 9V, 14, 18C, 19F and 23F[2]; PCV13 (Pfizer, USA) contains antigens for serotypes 1, 3, 4, 5, 6A, 6B, 7F, 9V, 14, 18C, 19A, 19F, and 23F and PCV7 (Pfizer, USA) against serotypes 4, 6B, 9V, 14, 18C, 19F, and 23F. Pooled vaccine efficacy to protect against invasive pneumococcal disease (IPD) caused by vaccine serotypes ranges from 71–93%, depending on schedule[2].

The World Health Organization (WHO) recommends vaccine schedules include two or three doses in the first year of life, with a booster at 9–15 months of age if using the two-dose schedule (3+0 or 2+1). If employing a three dose schedule in the first year of life, an optional booster after 12 months can be considered; however, it is noted that HIV+ infants can benefit from a booster dose in their second year of life. For children who did not receive PCV in the first year of life and as part of the national immunization schedule, the WHO recommends completing vaccination as part of a delayed or interrupted schedule for all children aged 12–24 months and children aged 2–5 years who are at high risk of pneumococcal infection [2].

PCV introduction across WHO Member States has begun in 70% of low-income, 64% of lower-middle income, and 45% of upper-middle income countries [3]. Coverage lags behind with Gavi, the Vaccine Alliance, estimating that PCV vaccine coverage of children was only 19% in countries it supports [4,5]. Consequently, many children eligible for PCV immunization in LMIC are not fully vaccinated before 12 months. Under-vaccination remains a problem even for traditional vaccines, leaving nearly 22 million children under 12 months of age unvaccinated each year. For example, a cross-sectional study in Ethiopia demonstrated that only 36.6% of children 12–23 months were fully vaccinated, and in an observational cohort study in rural Guinea-Bissau only half of infants were fully vaccinated by 12 months of age [6,7]. Low immunization coverage reduces potential indirect effects on adult morbidity and mortality from reduced nasopharyngeal carriage in paediatric populations [8]. One study in the US modeled a 54% reduction in nonbacteremic pneumococcal pneumonia in adults 65 years and older in states that had achieved over 80% vaccination coverage in children under two years [9]. In areas where vaccination coverage is low, more flexible immunization schedules are needed to enable the expanded coverage that can drive reductions in morbidity and mortality in both pediatric and adult populations.

Although the WHO recommends completing the immunization schedule for unvaccinated or not fully vaccinated children over 12 months, few countries are implementing this recommendation. Many of the LIC and LMIC have introduced PCV with funding from Gavi, which provides support for PCV administered in the first year of life, but not for older pediatric age groups. This is a risk to both children and their communities, as children under 5 years are at highest risk of contracting severe pneumonia as well as the most likely to transmit S. *pnuemoniae* [1, 10].

The effectiveness of extended age group vaccination with PCV in LMICs has not been explored. This systematic review examines the evidence base of the direct impact of extended age vaccination for children over 12 months with PCV in LMIC. Additional questions remain on the indirect effects of extended age group vaccination and would further complement this research.

## Methods

### Inclusion criteria

An a priori protocol was used and a PRIMSA checklist completed (S1 Checklist). Studies were included if they evaluated populations living in low- and middle-income countries that received their first dose of PCV between 12–60 months. The primary outcome measure was disease incidence with secondary outcome measures of antibody titers and nasopharyngeal carriage. All variations of extended age group vaccination were included, whether for vaccine introduction, catch-up campaigns, or routine extended age group vaccination. Reviews, models, editorials, and guidelines were excluded. The secondary effects of infant immunization on disease incidence in children were not explored. Language and date of publication were not exclusion criteria.

### Literature Search Strategy

The following databases were searched according to pre-specified search terms (S1 Table): PubMed (through October 21, 2013), LILACS (through November 4, 2013), Cochrane Infectious Diseases Group Specialized Register, Cochrane Central Register of Controlled Trials, CAB Abstracts (through November 5, 2013), and an abstract search from the 8^th^ and 9^th^ International Symposium on Pneumococci and Pneumococcal Diseases (ISPPD) was conducted March 25^th^, 2014. The abstract review and full-text review were conducted in duplicate with a third party reviewer for discrepancies- with the exception of the ISPPD abstracts, which were reviewed individually. An additional search across each of these databases was conducted November 16^th^, 2014 to identify recently published studies.

### Data Abstraction and Quality Appraisal

Two reviewers independently extracted authors, year of publication, study design, study population characteristics, intervention characteristics, comparators, outcome characteristics, and limitations. An assessment of the individual risk of bias in each of the studies was done in duplicate, using the Jadad scale for randomized control trials and the Newcastle Ottawa scales for observational studies, included for qualitative analysis [11, 12]. To assess the quality of evidence collected for each outcome measure, the framework established by the Grading of Recommendations Assessment, Development and Evaluation (GRADE) working group was used [13, 14].

### Data Synthesis

Data was synthesized based on three outcome measures: disease incidence, pneumococcal antibody titers, and nasopharyngeal carriage, with disease incidence selected as the primary outcome of interest. A logit transformation of the antibody response and variance were conducted; these were then weighted and pooled by serotype. When needed, authors were contacted for additional information.

## Results

**Fig 1:**
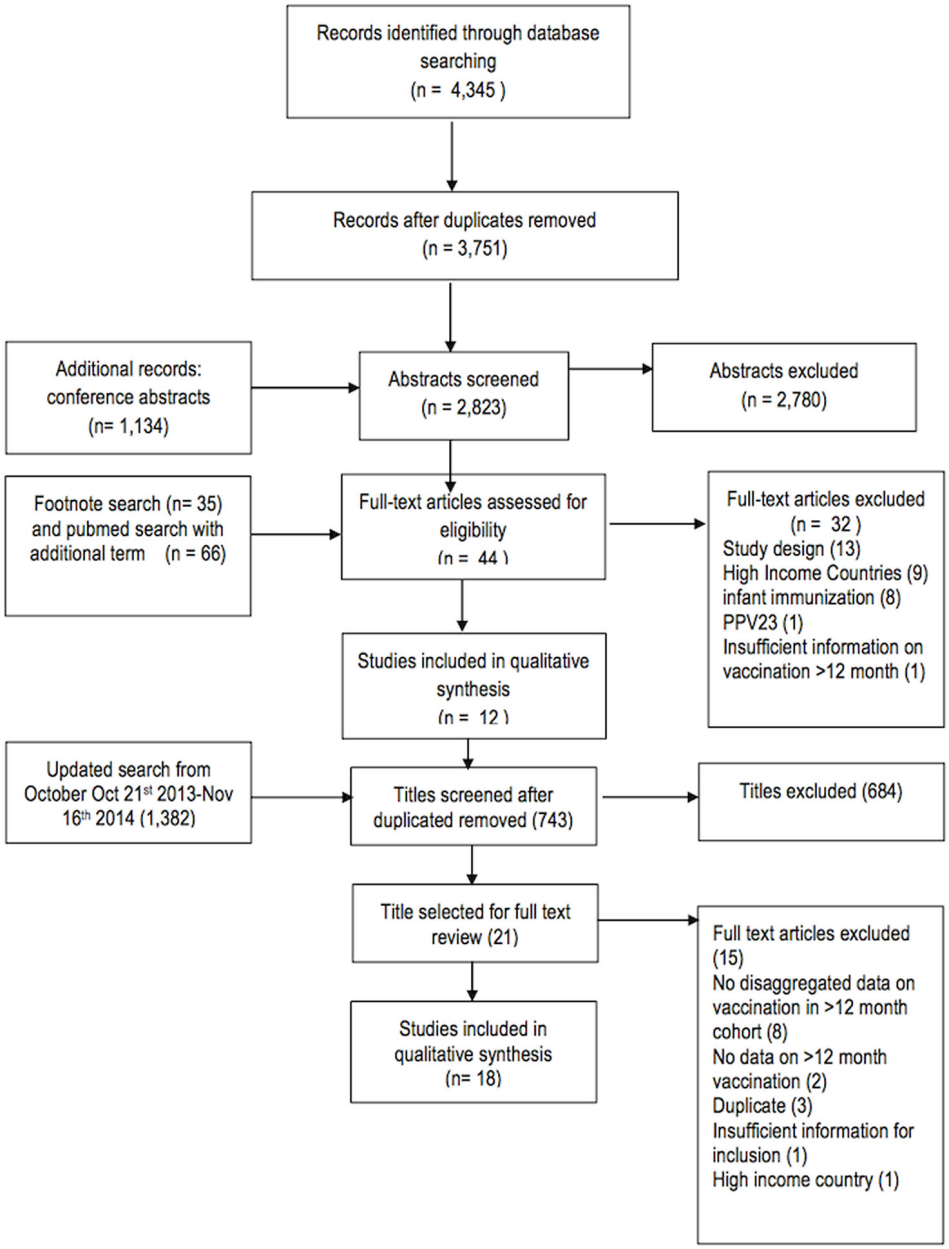
Search Strategy.

A total of 4,345 records were identified through the database search of which 594 were duplicates (Fig 1). An additional 1,134 abstracts were reviewed from the 8^th^ and 9^th^ ISPPD conference. 2,823 abstracts were selected for review in duplicate, resulting in 40 records selected for full-text review. A subsequent footnote search yielded an additional three studies for full-text review. Finally an expanded Pubmed search on an additional term (PHiD-CV) yielded 66 studies, of which one was eligible for full text review. Of the 44 records selected, 32 were excluded for the following reasons: study design (review, model, editorial) (13), High Income Countries (9), infant immunization (8), PPV23 (1) and insufficient data in vaccination over 12 months (1). An additional search across these databases from October 21^st^ 2013 to November 16^th^, 2014 yielded 1,382 additional studies, of which 639 were duplicates. The subsequent abstract search yielded 21 studies for full-text review, of which 15 were excluded due to lack of disaggregated data on children >12 months (8), duplicate study (3) no data on vaccination >12 months (2), high-income country (1) and insufficient data (1). The six remaining studies were added to the original 12 studies selected for inclusion in the systematic review, yielding 18 total studies.

Of the studies selected for inclusion, several provided data from the same site. An abstract and article from the same site in Brazil provided the same data on extended age group results and are reported together (Table 1) [15, 16]. Four studies present data from the same site in the Gambia over multiple years [17–20]. One included study reported on a control group where extended age group vaccination with PCV 7 was measured [21]. All of the studies reported on extended age group vaccination at the time of vaccine introduction.

**Table 1:** Characteristics of included studies.

Author	Year	Study design	Age*	Country	HIV+	Number	Intervention	Comparison	Outcome measure
Outcome: Disease incidence									
Domingues [15, 16]	2014	Matched case-control	1–2 years	Brazil	-	44	Catch-up with PCV 10	Unvaccinated children	68.0% effectiveness against IPD (95% CI 17.6–87.6%)
Pirez [31]	2011	Retrospective study	2–4 years	Uruguay	-	12,752	Catch-up with PCV 7	Children prior to vaccination	P-CAP discharge rates declined from 27.1% to 10.20% post immunization; CAP discharge rates declined from 15.6% to 7.0% post immunization
Outcome: Immune response									
de Carmago Costa [28]	2008	Cohort	2–9 years	Brazil	+	40	HIV+ children with PCV 7	No comparison	63.9% (95% CI 50.0–77.9%)) ≥1.3ug/ml for VT 39.3% (95% CI 25.6–53.0%) ≥ 4 fold increase in VT titre
Dicko [23]	2012	Cohort	1–2 years	Mali	-	69	Catch-up with PCV 10	No comparison	96.3% (95% CI 91.6–101.0%) ≥0.2ug/ml for VT
Dotres [21]	2014	RCT	4–5 years	Cuba	-	5	Safety and immunogenicity: PCV7	Children prior to vaccination	97.1% (95% CI 89.7–104.4%) (1 dose) ≥0.35ug/ml for VT 51.4% (95% CI 16.5–86.4%) (0 doses) ≥0.35ug/ml for VT
Hammitt [22]	2014	Double-blind RCT	1–5 years	Kenya	-	600	Catch-up with PCV 10	Unvaccinated children	91.0% (95% CI 88.8–93.2%) (1+ doses)≥0.35ug/ml for VT 97.2% (95% CI 95.8–98.7%) (2 doses) ≥0.35ug/ml for VT 30.2% (95% CI 24.1–36.3%) (0 doses) ≥0.35ug/ml for VT
Lagos [32]	2011	RCT	1–2 years	Chile	-	76	Catch-up with PCV 10	Children prior to vaccination	97.2% (95% CI 94.0–101.4%) (2 doses) ≥0.2ug/ml for VT 2.8% (95% CI 1.7–3.9%) (0 doses) ≥0.2ug/ml for VT
Lalwani [26]	2014	Randomized open label study	1–2 years	India	-	81	2 doses catch up with PCV 10	Children prior to vaccination	95.1% (95% CI 89.4–100.7%) (2 doses) > .2ug/ml for VT 11.0% (95% CI 3.9–18.2%) (0 doses) > .2ug/ml for VT
Odusanya [27]	2014	Open label controlled trial	1–2 years	Nigeria	-	35	2 doses catch-up with PCV 10	Children prior to vaccination	96.8% (95% CI 90–103.6%) (2 doses) > .2ug/ml for VT 14.0% (95% CI 2.2%-25.8%) (0 doses) > .2ug/ml for VT
Ota^Ɨ^ [18]	2012	RCT	2–4 years	The Gambia	-	44	Catch-up vaccination with PCV 7	Unvaccinated children	20.4% (95% CI 9.0–31.9%) (1 dose) ≥5.0ug/ml for VT
Thanee [29]	2011	Cohort	2–9 years	Thailand	+	89	HIV+ children receiving vaccine with PCV 7	Vaccinated HIV- children	94.8% (95% CI 88.5–101.0%) HIV- ≥0.35ug/ml for VT 94.2% (95% CI 88.9–99.5%) HIV+ ≥0.35ug/ml for VT 85.7% (95% CI 77.6–93.9%) HIV- ≥4 fold increase for VT 80.7% (95% CI 67.3–94.0%) HIV+ ≥4 fold increase for VT
Outcome: VT Carriage									
Roca^Ɨ^ [20]	2011	RCT	2–5 years	The Gambia	-	219	Catch-up with PCV 7	Unvaccinated children	OR of VT carriage 0.213, SE 3.69
Roca^Ɨ^ [19]	2012	RCT	2–4 years	The Gambia	-		Catch-up with PCV 7	Unvaccinated children	VT mean density of carriage by 28.1% (2.87 vs 2.48), control population 13.5% decline (2.70 vs 1.94)
Roca^Ɨ^ [17]	2013	RCT	2–4 years	The Gambia	-	783	Catch-up with PCV 7	Unvaccinated children	Carriage prevalence 13.6% (12/88) partially vaccinated; 8.9% (10/112) wholly vaccinated. OR = 0.69 (0.20, 2.32)
Andrade [30]	2014	Case-control study	1–2 years	Brazil	-	311	Catch-up with PCV 10	Unvaccinated children	Risk ratio 0.88 (95% CI 0.556–1.120) of pneumococcal vaccine-type carriage,
Hammitt [24]	2014	Before and after study	1–4 years	Kenya	-	107	2 doses catch up with PCV 10	Children receiving 0 or 1 doses of PCV	Prevalence ratio 0.47 (95% CI 0.21–1.03) for vaccine serotype pneumococci
Makenga [25]	2014	Crossover	1–5 years	Tanzania	+	73	Catch-up vaccination with PCV 13	Unvaccinated children	Overall pneumococcal isolation rate at baseline was 71% (n = 73) and 73% (n = 68) after two doses

*Age at vaccination

^Ɨ^ Data obtained from same study site

Characteristics of included studies are listed (Table 1). Eight of the studies were conducted in low income countries [17–20, 22–25], two in lower middle income countries [26–27], six in upper middle income countries [15, 16, 21, 28–30], and two in high income countries [31, 32], though they were classified as upper middle income countries at the time of the study. These studies were published between 2008 and 2014, with three presented as abstracts at the ISPPD [15, 23, 25]. Eight studies report on 1 dose of PCV catch up [15–21, 30] and eight report on 2 doses of PCV catch up [23–28, 31, 32], with two reporting both one and two dose extended age regimens [22, 29]. Nine studies were from Africa [17–20, 22–25, 27]^,^ seven from South America [15, 16, 21, 28, 30–32], and two from Asia [26, 29]. Eight examined PCV 7[17–21, 28–29, 31], nine examined PCV 10 [15, 16, 22–24, 26, 27, 30, 32], and one examined PCV 13 [25]. Three reported on disease incidence (though two report on the same data and are combined)[15, 16, 31], six reported on nasopharyngeal carriage [17, 19, 20, 24, 25, 30], and nine on immune response [18, 21–23, 26–29, 32] (Table 1). A GRADE analysis indicated that the studies were of high quality (S2 Table). Meta-analysis was not possible due to the heterogeneity of outcome measures with the exception of pooled immune response by threshold and serotype.

### Primary Outcome: Disease Incidence

#### Disease incidence: IPD

Three studies reported on disease incidence, with all reporting significant efficacy in either the most prevalent serotype or across serotypes [15, 16, 31]. A matched case-control study in Brazil showed a 68% VE (95% CI 17.6–87.6%) for VT-IPD for children immunized with a single dose of PCV10 as catch up between 12–23 months; these results are reported in two of the studies that have been included [15, 16].

#### Disease incidence: Pneumonia

One study used hospital discharge rates for pneumococcal community-acquired pneumonia (P-CAP) before and after PCV7 introduction, including catch up for children 12–24 months, as a proxy for disease incidence [31]. In this study, coverage of catch up population was high with 85% for the first dose and 70% for the second dose. P-CAP hospital discharge rates for children 2–4 years of age (who were over 12 months of age at the time of vaccine introduction) declined from 8 per 10,000 pre vaccination to 2 per 10,000 post-PCV7 roll-out (not significant), though significant declines from 17.6 (95% CI 11.4–25.9) to 5.1(95% CI 2.8–10.2) per 10,000 were detected in serotype 14, the serotype most prevalent in that context [31].

### Secondary outcome: immune response

In the nine studies reporting on immune response, five different thresholds for immune response were used, with most studies reporting on multiple thresholds: ≥0.2μg/ml, ≥0.35μg/ml, ≥1.3μg/ml, ≥5μg/ml and ≥4-fold increase [18, 21–23, 26–29, 32]. These thresholds measure the proportion of children achieving various levels of immune response to vaccine serotypes. The ≥0.35μg/ml thresholds and ≥0.2μg/ml threshold have been reported together when the latter threshold is tested with a 22F polysaccharide inhibition ELISA, due to the improved specificity [33]. The ≥1.3μg/ml threshold and ≥4-fold increase in titres were selected by one study as surrogate markers for immune response for immunosuppressed children [28]. An immune response threshold over ≥5μg/ml was selected as a measure of carriage protection [18].

**Fig 2:**
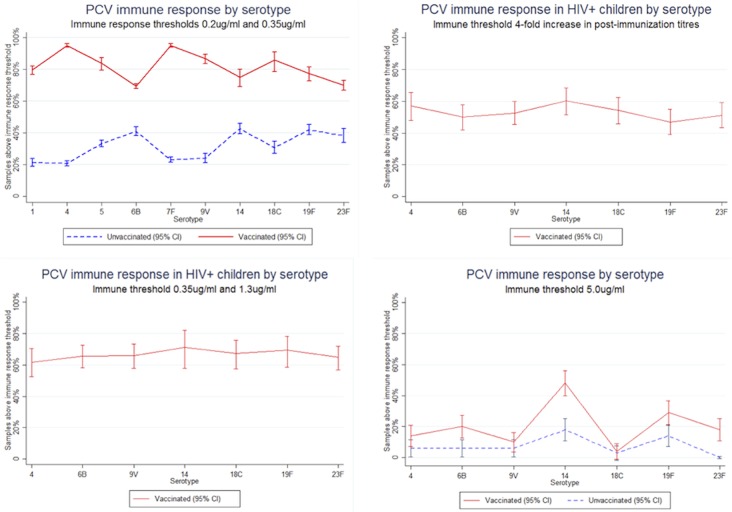
PCV immune response by serotype.

Significant immune response was detected across all serotypes at the standard threshold >0.2μg/ml and >0.35μg/ml for vaccinated versus unvaccinated subjects (Fig 2). Among the vaccinated, immune response ranged from 69.0% (95% CI 64.4–73.6%) for serotype 23B [22] to 100% in 30 of the 74 immune responses reported by studies included in this systematic review. For unvaccinated children, immune response ranged from 1.40% (95% CI 0.0–7.4%) [32] for serotype 6B and 43.0% (95% CI 36.1–50.0%) for serotype 14 [22].

Two studies reported on immune response to VT serotypes among HIV+ children 2–9 years [28, 29]. One study found that 94.2% (95% CI 88.9–99.5%) of children achieve immune responses above the 0.35μg/ml threshold, similar to the 94.8% (95% CI 88.5–101.0%) [29] immune response for HIV negative children at the same threshold [29]. When the threshold was elevated to 1.3μg/ml threshold, 63.9% (95% CI 50.0–77.9%) of children surpassed the threshold, and when it was further elevated to 4-fold increase in post immunization titres, 53.2% (95% CI 45.0–61.2%) achieved a 4-fold increase in post immunization titres [28]. These additional thresholds were incorporated as surrogate markers of adequate immune response for an immunocompromised population [28].

At the carriage protection threshold (>5.0ug/mL), the one study reporting this data found significantly higher protection in vaccinated groups compared to unvaccinated in serotypes 6B, 14, 19F and 23F [18].

### Tertiary outcome: carriage

Six studies reported on carriage at different time points [17, 19, 20, 24, 25, 30]. Nasopharyngeal carriage showed significant declines across studies from the Gambia [17, 19] where children over 30 months of age were vaccinated with PCV7 in intervention villages and were not vaccinated in control villages. Children less than 30 months in all villages were also vaccinated. In children under five years, pneumococcal carriage declined by 28.1% for VT serotypes in vaccinated villages compared to a 13.5% decline in unvaccinated villages [19]. The OR of carriage in intervention villages receiving extended age group vaccination was 0.213 compared to control villages only receiving the vaccine up to 30 months [20]. Even four years after PCV introduction, decreases in vaccine carriage in interventions persisted with an OR of carriage of 0.69 compared to control villages in children under five years [17]. A cross sectional study in Brazil examined nasopharyngeal carriage of PCV10 serotype pneumococcus in 311 children who received a single dose of PCV10 between 12–23 months. As compared to the unvaccinated, this group had a rate ratio of 0.820 (95% CI 0.556–1.210), which was not significant [30]. A cross-sectional analysis from Kenya found a 0.47 (95% CI 0.21–1.03) prevalence ratio of vaccine type pneumococci prevalence in children 1–4 receiving 2 PCV 10 doses administered after 1 year of age, compared to children 1–4 who received zero or one dose of PCV 10 [24]. The one study reporting on pneumococcal isolation rate from nasopharyngeal swabs in Tanzania found high rates of serotype replacement in HIV+ children with a non-significant increase in pneumococcal isolation rates from 71% to 73% after doses administered after 1 year of age [25].

## Discussion

While the studies examined three different outcomes, the trend of each was clear: administering PCV to children over 12 months in LMICs shows significant impact. With 21.8 million children completing even the basic package of recommended immunizations each year, policies that restrict PCV immunization to children under 12 months will impact a significant portion of the birth cohort and will lead to consistently low PCV coverage rates even years after vaccine introduction [34]. The children who miss the basic immunization schedule are systematically more disenfranchised and vulnerable than the children receiving immunizations within the first year [5]. This includes children living in humanitarian emergencies particularly at-risk for IPD [35, 36] and where the large-scale population upheaval disrupts often-fragile routine immunization structures, resulting in more children in need of vaccination services beyond their first year. Organizations such as MSF are beginning to use PCV in emergencies and refugee camps, including for use in children over 12 months.

While this study does not systematically examine the indirect effects of extended age group vaccination on the population, these effects may further accentuate the public health benefit of extended age group vaccination. As recent evidence indicates that 66% coverage in children under five years is sufficient for herd immunity, extending the window for vaccinating children can provide population-wide benefits [37]. However, as many communities in low- and middle-income countries do not achieve standard EPI vaccination coverage of even 50% by 12 months, benefits from herd immunity may not be realized if the schedule for delayed and interrupted immunization above 12 months is not supported [37].

As one of the most expensive vaccines recommended by WHO for inclusion in routine immunization, the cost of PCV can be prohibitive, discouraging countries from including it in their EPI schedules, especially for older age groups when there is a lack of donor support. While price data from pharmaceutical companies is limited, publicly available information shows PCV is sold at USD$3.30-$7 per dose purchased through Gavi; USD$14.12-$15.84 per dose to the Pan American Health Organization (PAHO) Revolving Fund; and USD$116.91 per dose to the US government [38–40]. Non-governmental actors, such as humanitarian organizations or other non-profit health service providers, may not be able to access lower prices such as those negotiated by Gavi. Finally, as donor support from organizations such as Gavi is limited to PCV vaccination for those less than 12 months, countries alone may not be able to shoulder the burden of providing catch up vaccination.

Given the large and significant impact of PCV administration in older age groups in LMICs, countries should change national guidelines to reflect the need for extended age group vaccination. In its support of national EPI programmes, Gavi and its donors should respond to WHO recommendations and countries’ needs and expand the vaccine subsidy window for vaccination in children up to age five. Additionally, policy should be formulated to ensure that PCV is used in emergency contexts, including in extended age groups, as a rapid intervention to limit IPD-related morbidity and mortality. Concurrently, the global immunization community–including Gavi, but also humanitarian actors such as OCHA, UNHCR, UNICEF, WHO and NGOs–should address the obstacles to systematically using PCV as part of the health service package in emergencies.

### Limitations

Study constraints exist, including a limited number of studies examining disease incidence in extended age groups, few comparative study designs, small sample sizes in several studies, and differing age ranges reported. As extended age group vaccination has not been widely implemented, the included studies report only on catch-up immunization at vaccine introduction. As the WHO recommendations for extended age vaccination are implemented, further quantification of indirect effects of extended age group vaccination can be undertaken. Additionally the lack of standard immune response thresholds for children 12–59 months and for HIV positive children force assumptions on appropriate vaccine immunity thresholds.

## Conclusions

Vaccination with PCV in extended age groups is effective in LMIC across a range of outcomes. In light of this evidence, countries should review and revise national guidelines to reflect WHO recommendations. Donors should extend support to Gavi-eligible countries and humanitarian organizations for implementing extended age group vaccination. More research on disease incidence can determine the most appropriate context and schedules for extended age group immunization.

## Supporting Information

S1 Checklist PRISMA 2009 Checklist. (DOC)

S1 Table Search terms. (DOC)

S2 Table Assessments of bias and GRADE Analysis. (DOCX)
