# Supplementary material for: Impact of Pneumococcal Conjugate Vaccine Administration in Pediatric Older Age Groups in Low and Middle Income Countries: A Systematic Review
Source: PLoS One. 2015 Sep 2;10(9):e0135270. doi: 10.1371/journal.pone.0135270 (PMC4557974; doi:10.1371/journal.pone.0135270)
Supplement: S1 Table — (DOC) [file pone.0135270.s002.doc]

S1 Table

Search Strategy

**Search 1: PubMed October 21, 2013**

1. streptococcus pneumonia OR pneumococcus OR pneumococcal OR pneumococcal conjugate vaccine
2. pneumococcal conjugate vaccine OR conjugate vaccines OR pneumococcal vaccine
3. surveillance OR carrier state OR nasopharyngeal carriage OR immunogenicity OR pneumococcal infections OR streptococcus pneumoniae infections OR pneumococcal pneumonia OR streptococcus pneumoniae pneumonia OR pneumococcal meningitis OR streptococcus pneumonia meningitis OR vaccine efficacy OR childhood mortality OR child mortality OR infant mortality

Search #1 AND #2 AND #3

Yields 3364 (minus 13 duplicates within PubMed) = 3351 results

388 titles identified for an abstract search.

**Search 2: LILACS November 4, 2013**

1. tw: (streptococcus pneumonia OR pneumococcus OR pneumococcal OR pneumococcal conjugate vaccine)
2. tw: (pneumococcal conjugate vaccine OR conjugate vaccines OR pneumococcal vaccine)
3. tw: (surveillance OR carrier state OR nasopharyngeal carriage OR immunogenicity OR pneumococcal infections OR streptococcus pneumoniae infections OR pneumococcal pneumonia OR streptococcus pneumoniae pneumonia OR pneumococcal meningitis OR streptococcus pneumonia meningitis OR vaccine efficacy OR childhood mortality OR child mortality OR infant mortality)

Search #1 AND #2 AND #3

Yields 8 results

4 PubMed duplicates = 4 new results

3 titles identified for an abstract search.

**Search 3: Cochrane Infectious Diseases Group Specialized Register November 5, 2013**

1. vaccine

Yields 15 results

*Note: searching only “pneumonia” or only “pneumococcal” yields 0 results.

0 titles identified for an abstract search.

**Search 4: Cochrane Central Register of Controlled Trials November 5, 2013**

1. streptococcus pneumonia OR pneumococcus OR pneumococcal OR pneumococcal conjugate vaccine
2. pneumococcal conjugate vaccine OR conjugate vaccines OR pneumococcal vaccine
3. surveillance OR carrier state OR nasopharyngeal carriage OR immunogenicity OR pneumococcal infections OR streptococcus pneumoniae infections OR pneumococcal pneumonia OR streptococcus pneumoniae pneumonia OR pneumococcal meningitis OR streptococcus pneumonia meningitis OR vaccine efficacy OR childhood mortality OR child mortality OR infant mortality

Search #1 AND #2 AND #3

Yields 335 results (minus 5 duplicates within CENTRAL) = 330 results

256 duplicates between CENTRAL and PubMed = 74 new results

25 titles identified for an abstract search.

**Search 5: CAB Abstracts November 5, 2013**

1. streptococcus pneumonia OR pneumococcus OR pneumococcal OR pneumococcal conjugate vaccine
2. pneumococcal conjugate vaccine OR conjugate vaccines OR pneumococcal vaccine
3. surveillance OR carrier state OR nasopharyngeal carriage OR immunogenicity OR pneumococcal infections OR streptococcus pneumoniae infections OR pneumococcal pneumonia OR streptococcus pneumoniae pneumonia OR pneumococcal meningitis OR streptococcus pneumonia meningitis OR vaccine efficacy OR childhood mortality OR child mortality OR infant mortality

Search #1 AND #2 AND #3

Yields 129 results.

66 duplicates with PubMed and 2 duplicates with CENTRAL = 61 new results.

28 titles identified for an abstract search.

**Search 6: EMBASE November 5, 2013**

1. streptococcus pneumonia  OR pneumococcus OR pneumococcal OR pneumococcal conjugate vaccine
2. pneumococcal conjugate vaccine OR conjugate vaccines OR pneumococcal vaccine
3. surveillance OR carrier state OR nasopharyngeal carriage OR immunogenicity OR pneumococcal infections OR streptococcus pneumoniae infections OR pneumococcal pneumonia OR streptococcus pneumoniae pneumonia OR pneumococcal meningitis OR streptococcus pneumonia meningitis OR vaccine efficacy OR childhood mortality OR child mortality OR infant mortality

Search #1 AND #2 AND #3

Yields 509 results.

250 duplicates with the other databases = 259 new results.

111 titles identified for an abstract search.

**Search 7: ISPPD April 2014**

ISPPD 2012: Identified 24 abstracts (618 total), 2 selected for inclusion (neither had developed into papers)

ISPPD 2014: Identified 23 abstracts (553 total), 3 selected for inclusion

**Search 8: Pubmed March 21^st^, 2014**

- In the abstract review, I found repeated references to PHiD-CV (Another term for PCV 10). This was not used in the original systematic review search criteria. With just a brief search of PHid-CV on pubmed (only yield 64 studies)

**Search 9: Updated terms for Pubmed, EMBASE , CABS abstracts, LILACS, Cochrane infectious disease**

Pubmed Oct 21^st^ 2013-Nov 16^th^ 2014

522 studies retrieved

EMBASE Nov 16^th^ 2014

573 selected- but included all studies- when studies before Oct 2013 were deleted, 108 remained

108 remained from 2013-2014

LILACS

Yielded 0 results

CABS abstracts

Searched 2013-current (Nov 16^th^ 2014)

Identified 150 abstracts

Among CABS, LILACS, EMBASE and pubmed 1382 were identified- 639 duplicate references were deleted, leaving 743 remaining

Cochrane database (searched ‘vaccine’)

25 studies from Oct 2013 to Nov 16^th^, 2014

Cochrane register of studies

194 selected- none are relevant in the Nov 5^th^ 2013- Nov 16^th^ 2014 time window
