# Supplementary material for: Impact of Pneumococcal Conjugate Vaccine Administration in Pediatric Older Age Groups in Low and Middle Income Countries: A Systematic Review
Source: PLoS One. 2015 Sep 2;10(9):e0135270. doi: 10.1371/journal.pone.0135270 (PMC4557974; doi:10.1371/journal.pone.0135270)
Supplement: S2 Table — (DOCX) [file pone.0135270.s003.docx]

|  |  | **Pirez** | **Dicko** | **Costa** | **Domingues** | **Lagos** | **Thanee** | **Andrade** | **Hammitt** | **Odusanya** | **Lalwani** |
| --- | --- | --- | --- | --- | --- | --- | --- | --- | --- | --- | --- |
| **Selection** | **Representativeness of the exposed cohort** | ***** | **0** | **0** | ***** | **0** | **0** | ***** | ***** | **0** | ***** |
|  | **Selection of the non-exposed cohort** | ***** | **0** | **0** | ***** | **0** | ***** | ***** | ***** | **0** | ***** |
|  | **Ascertainment of exposure** | ***** | ***** | ***** | ***** | ***** | ***** | ***** | ***** | ***** | ***** |
|  | **Demonstration that outcome of interest was not present at start of study** | ***** | ***** | ***** | ***** | ***** | ***** | ***** | ***** | **0** | ***** |
| **Comparability** | **Comparability of cohorts on the basis of the design or analysis** | ***** | **0** | **0** | **2** | **0** | ***** | **2** | ***** | ***** | ***** |
| **Outcome** | **Assessment of outcome** | ***** | ***** | ***** | ***** | ***** | ***** | ***** | ***** | ***** | ***** |
|  | **Was follow-up long enough for outcomes to occur** | ***** | ***** | ***** | **0** | **0** | **0** | **0** | **0** | ***** | ***** |
|  | **Adequacy of follow-up of cohorts** | ***** | **0** | ***** | **0** | **0** | **0** | **0** | **0** | ***** | ***** |
|  |  | **8/9** | **4/9** | **5/9** | **7/9** | **5/9** | **7/9** | **6/9** | **6/9** | **5/9** | **8/9** |

Appendix S2. Assessments of bias and GRADE Analysis

Newcastle Ottawa

Jadad

| **Item** | **Description** | **Makenga 2014** | **Ota 2012** | **Roca 2011** | **Hammitt 2014** | **Roca 2012** | **Roca 2013** | **Dotres 2014** |
| --- | --- | --- | --- | --- | --- | --- | --- | --- |
| **Randomization** | 1 point if randomization is mentioned | 1 | 1 | 1 | 1 | 1 | 1 | 1 |
|  | 1 additional point if the method of randomization is appropriate | 1 | 1 | 1 | 1 | 1 | 1 | 0 |
|  | Deduct 1 point if the method of randomization is inappropriate | 0 | 0 | 0 | 0 | 0 | 0 | 0 |
| **Total Randomization** |  | 2 | 2 | 2 | 2 | 2 | 2 | 1 |
| **Blinding** | 1 point if blinding is mentioned | 1 | 1 | 1 | 1 | 1 | 1 | 1 |
|  | 1 additional point if the method of blinding is appropriate | 1 | 1 | 1 | 1 | 1 | 1 | 1 |
|  | Deduct 1 point if the method of blinding is inappropriate | 0 | 0 | 0 | 0 | 0 | 0 | 0 |
| **Total Blinding** |  | 2 | 2 | 2 | 2 | 2 | 2 | 2 |
| **An account of all patients** | The fate of all patients in the trial is known. If there are no data the reason is stated. | 0 | 0 | 0 | 1 | 0 | 0 | 1 |
| **Total Overall** |  | 4 | 4 | 4 | 5 | 4 | 4 | 4 |

GRADE analysis

|  |  |  |  |  | | Decrease GRADE | | | | | Increase GRADE | | |  |  |
| --- | --- | --- | --- | --- | --- | --- | --- | --- | --- | --- | --- | --- | --- | --- | --- |
| Comparison | Outcome | Quantity and Type of Evidence | Findings | Starting GRADE | | Study quality | Consistency | Directness | Precision | Publication Bias | Large Magnitude | Dose-response | Confounder | GRADE of Evidence for Outcome | Overall GRADE of Evidence Base |
| What is the impact of vaccinating older pediatric age groups with PCV in low- and middle-income countries? | | | | | | | | | | | | | | | |
| PCV in 12-60 months vs. no PCV | IPD* | 2 Case-control, 1 Retrospective cohort | Domingues, Domingues , Hammitt, Pirez | | Low | 0 (balance each other out--low vs high sample sizes | 0 |  | 0 (balance each other out--low vs high sample sizes) | -1 (cannot exclude publication bias) | (+)1 | (+)1 (two doses shown as better than 1) | 0 | high | high |
|  | Antibody titers | 5 randomized controlled trials, 4 cohort | Costa, Dicko, Dotres, Hammitt, Lagos, Lalwani, Odusanya,Ota, Thanee | | High |  | 0 |  | 0 | -1 (cannot exclude publication bias) | (+)1 | 0 | 0 | high | high |
|  | Nasopharyngeal Carriage | 4 cluster RCT, 1 before and after crossover | Roca, Roca, Roca, Andrade, Makenga | | High | 0 | 0 | -1 (PCV7) | 0 | -1 (cannot exclude publication bias) | (+)1 | 0 | 0 | high | high |

*Critical outcome
